# Evaluation of inpatients *Clostridium difficile* prevalence and risk factors in Cameroon

**DOI:** 10.1186/s40249-020-00738-8

**Published:** 2020-08-31

**Authors:** Ingrid Cécile Djuikoue, Ernest Tambo, Gildas Tazemda, Omer Njajou, Denise Makoudjou, Vanessa Sokeng, Morelle Wandji, Charlène Tomi, Aubain Nanfack, Audrey Dayomo, Suzie Lacmago, Falubert Tassadjo, Raissa Talla Sipowo, Caroline Kakam, Aicha Bibiane Djoko, Clement Nguedia Assob, Antoine Andremont, Frédéric Barbut

**Affiliations:** 1 Département de Microbiologie de la Faculté des Sciences de la Santé de l’Université des Montagnes, Bangangte, Cameroon; 2 Prevention and Control Foundation, Bangangte, Cameroon; 3 Medical Diagnostic Centerto Yaounde, Yaounde, Cameroon; 4 Laboratoire de Bactériologie du Centre Pasteur du Cameroun, Yaounde, Cameroon; 5 Faculty of Health Sciences, University of Buea, PO Box 63, Buea, SW Region Cameroon; 6 Faculty of Medicine, Xavier-Bichat Campus, University of Paris VII – Denis Diderot, Paris, France; 7 Faculty of Pharmacy, Paris – University of Paris Descartes, Paris, France

**Keywords:** *Clostridium difficile*, Prevalence, Diarrhea, Risk factors, Quinolone, Cephalosporin, Cameroon

## Abstract

**Background:**

*Clostridium difficile*, rarely found in hospitals, is a bacterium responsible for post-antibiotic diarrhea and Pseudomembranous Colitis (CPM). *C. difficile* selective pressure represents potential public health problem due to the production of toxins A and B serious pathologies effects/consequences. A transversal and analytic study was to assess the risk factors of *C. difficile* infection and to determine the prevalence of *C. difficile* in patients received in randomly selected five hospitals in Yaoundé, Cameroon.

**Methods:**

A total of 300 stool samples were collected from consented patients using a transversal and analytic study conducted from 10th July to 10th November 2018 in five hospitals in Cameroon. The detection or diagnostic kit was CerTest *C. difficile* Glutamate Dehydrogenase + Toxin A + Toxin B based on immuno-chromatographic assay. A univariate and multivariate analysis allowed us to highlight the associated factors.

**Results:**

The results showed a prevalence of *C. difficile* of 27.33% (82/300 stool patients’samples taken). Of these 27.33%, the production of Toxin A and Toxin B were 37.80 and 7.31% respectively. In univariate analysis, hospitalization was a significant (*P* = 0.01) risk factor favoring *C. difficile* infection. In multivariate analysis, corticosteroids and quinolones use/administration were significantly (adjusted Odd Ratio, a*OR* = 14.09, 95% *CI*: 1.62–122.54, *P* = 0.02 and a*OR* = 3.39, 95% *CI*: 1.00–11.34, *P* = 0.05 respectively) risk factor for this infection.

**Conclusion:**

The prevalence of *C. difficile* infections (CDI) remain high in these settings and may be related not only to permanent steroids and antibiotics. Promoting education to both medical staff and patients on the prevalence and public health impact of *C. difficile* can be core inimproving rationale prescription of steroids and antibiotics to patients and promote human health and exponential growth in Cameroon.

## Background

*Clostridium difficile* is the most frequent infectious cause of nosocomial diarrhea and a major financial burden for health-care systems [1–3]. It is responsible of about 25% of reported antibiotic-associated diarrhea cases and virtually all cases of pseudomembranous colitis (PMC) worldwide [4]. The clinical spectrum of *C. difficile* infection (CDI) varies in severity from asymptomatic carriage, to self-limited, mild, watery diarrhea, intestinal perforation, toxic megacolon, sepsis, fulminant colitis, and death [3]. Major CDI risks factors reported include the antibiotic use, advanced age, and unsafe exposure to healthcare facilities. The epidemiology of CDI has changed since the emergence of the *C. difficile* 027/NAP1/BI strain, which has been implicated in large outbreaks, with a notable increase in the incidence and severity of the disease [5].

There are scarcity of data published and information sharing platforms on CDI epidemiology in sub-Sahara Africa [6, 7]. Little is documented on the risk factors and prevalence of *C. difficile* in hospitals and community health care settings in Cameroon. Understanding and identifying patients at risk of CDI is an important step in care delivery decision making process and infection management to resistance disease prevention and control. The study aims at defining the risk factors of CDI, the prevalence and impact of treatment outcomes in order to contribute to the fight against *C. difficile* resistance emergence in Cameroon.

## Methods

### Study site and population

A transversal and analytic study was executed from 10th July to 10th November 2018 in five hospitals, namely, CHUY: Centre Hospitalier et Universitaire de Yaoundé; HCY: Hôpital Central de Yaoundé; HM: HôpitalMilitaire; HP: Hôpital de Police; HE: Hôpital d’Efoulan. After an approved informed consent by the patients, a pretested questionnaire was administered to collect sociodemographic and clinical information and vital signs data (Table 1).

**Table 1:** Hospital-based distribution of participants, Yaoundé, Cameroon (*N* = 300)

Sites	***n*** (Proportion, %)
University Hospital Center, Yaoundé (CHUY)	96 (32.0)
Central Hospital, Yaoundé (HCY)	74 (24.7)
Military Hospital (HM)	53 (17.7)
Police Hospital (HP)	42 (14.0)
Efoulan Hospital (HE)	35 (11.7)
**Sexe**	***n*** **(%)**
Female	145 (48.3)
Male	155 (51.7)
**Hospitalization**	***n*** **(%)**
Not	108 (36.0)
Yes	192 (64.0)
**HIV**	***n*** **(%)**
Not	196 (65.3)
Yes	104 (34.7)
**Use of penicilline**	***n*** **(%)**
Not	281 (93.7)
Yes	19 (6.3)
**Use of cephalosporine**	***n*** **(%)**
Not	288 (96.0)
Yes	12 (4.0)
**Use of imidazole**	***n*** **(%)**
Not	280 (93.3)
Yes	20 (6.7)
**Use of aminoside**	***n*** **(%)**
Not	296 (98.7)
Yes	4 (1.3)
**Use of vancomycine**	***n*** **(%)**
Not	295 (98.3)
Yes	5 (1.7)
**Use of quinolone**	***n*** **(%)**
Not	289 (96.3)
Yes	11 (3.7)
**Use of corticoids**	***n*** **(%)**
Not	294 (98.0)
Yes	6 (2.0)

### Sampling and inclusion criteria

A cross sectional study was performed and samples were processed at Medical Diagnostic Center Laboratory, Yaoundé, Cameroon. Our population was made up of inpatients admitted over 48 h, community patients aged up 21 to 88 years old, suspected of CD infection. Inpatients that consented and met the inclusion criteria, participated into the study. Add the exclusion criteria.

### Data collection and quality control

Data were collected from enrolled patients in the five selected hospitals (Yaoundé Hospital and University Center, Yaoundé Central Hospital, Military Hospital, Police Hospital and Efoulan District Hospital) in Yaoundé city, Cameroon. A pretested questionnaire including age, gender, comorbid, hospital admission time, signs and symptoms of CDI and duration of the illness, CDI risk factors, health history, use of antibiotics and antiviral drugs, immunosuppessors, antihistaminic drugs antagonists, antacid and proton pump inhibitors or antiperistalsis and surgery methods were documented. Antibiotics were grouped into and others.

### Microbiological assays

Fecal samples were collected, stored and analyzed at Medical Diagnostic Center Laboratory, Yaoundé. The presence of *C. difficile*, was evaluated on these fresh samples using immunochromatographic test kit detecting Glutamate dehydrogenase toxins A/B (C. DIFF QUIK CHEK®) Roche, Paris, France [8].

### Data analysis

StartView statistical software, Version 23 was used to process and analyze the data chi-square and Fisher tests were used to evaluate and establish the correlations between variables, GDH enzyme, toxins A and B. *P* value less than 0.05 was considered as statistically significant.

### Ethical statement

The ethical approval was received from ethical Review Board of Higher Institute of Health Sciences, Universite des Montagnes and local authoritiespermission in the various hospitals in Yaoundé, Cameroon.

## Results

### General characteristics of the study population

A total of 300 patients were enrolled including 108 admitted and 192 outpatients in the selected five hospitals in Yaoundé. The mean age was 32.7 ± 17.3 years old (range 1–88 years old). The gender ratio was 1.071 (male:female). The number of patients according to hospital site were 96 patients (32.0%) were CHUY, 74 patients (24.7%) on HCY, 53 patients (17.7%) on HM, 42 patients (14.0%) on HP and 35 patients (11.7%) on HE **(**Table 1**).**

### Analysis of risk factors of CDI reservoirs based on antibiotic classes

A total of 82 (27.33%) were positive; 31(37.8%) secreted toxin A and 06 (7.3%) toxin A + B. Using univariate analysis, we found that quinolones and corticoids were significantly and independently associated with *C. difficile* infection (*P* = 0.05 & 0.02) respectively. Whereas, a multivariate logistic regression analysis showed that corticoids use/uptake (a*OR* = 14.0; 95% confidence interval [*CI*]: 1.62–122.54; *P* = 0.02), quinolones (a*OR* = 3.39; 95% *CI*: 1.00–11.34; *P* = 0.05) and hospitalization (a*OR* = 2.10; 95% *CI*: 1.18–3.72; *P* = 0.12) were significantly associated with CDI in these hospitals in Cameroon (Table 2**).**

**Table 2:** Prevalence of *Clostridium difficile* infections according to age, sex, hospital, hospitalization, HIV status, antibiotic class and corticoid treatment

Variables	Absence of ***C. difficile n*** (%) / mean	Presence of ***C. difficile n*** (%) / mean	Brute ***OR***	95% ***CI***	***P***-value
**Age**					
Age ± *SD*	33.72 ± 2.3	30.12 ± 3.7	0.99	0.97–1.00	0.11
**Sex**					
Female	104 (71.7)	41 (28.3)	1		
Male	114 (73.5)	41 (26.4)	0.91	0.55–1.52	0,72
**Hospital**					
CHUY	72 (75.0)	24 (25.0)	1		
HCY	50 (67.6)	24 (32.4)	1.44	0.74–2.82	0.29
HM	35 (66.0)	18 (33.9)	0.89	0.74–3.21	0.25
HP	34 (80.9)	8 (19.0)	1.54	0.29–1.73	0.45
HE	27 (77.1)	8 (22.8)	0.71	0.36–2.22	0.80
**Hospitalization**					
Not	88 (81.5)	20 (18.5)	1		
Yes	130 (67.7)	62 (32.2)	2.10	1.18–3.72	0,01
**HIV**					
Not	139 (70.9)	57 (29.0)	1		
Yes	79 (75.9)	25 (24,0)	0.77	0.4–1.33	0.35
**Use of penicilline**					
Not	204 (72.6)	77 (27.4)	1		
Yes	14 (73.7)	5 (26.3)	0.95	0.33–2.72	0.92
**Use of cephalosporine**					
Not	209 (72.6)	79 (27.4)	1		
Yes	9 (75.0)	3 (25.0)	0.88	0.23–3.34	0.85
**Use of imidazole**					
Not	203 (72.5)	77 (27.5)	1		
Yes	15 (75.0)	5 (25.0)	0.88	0.31–2.5	0.81
**Use of aminoside**					
Not	216 (72.9)	80 (27.0)	1		
Yes	2 (50.0)	2 (50.0)	2.7	0.37–19.5	0.33
**Use of vancomycine**					
Not	216 (73.2)	79 (26.8)	1		
Yes	2 (40.0)	3 (60.0)	4.10	0.67–27.0	0.13
**Use of quinolone**					
Not	209 (72.3)	80 (27.7)	1		
Yes	9 (81.8)	2 (18.2)	0.58	0.12–2.75	0.49
**Use of corticoids**					
Not	214 (72.8)	80 (27.2)	1		
Yes	4 (66.66)	2 (0.3)	1.34	0.24–7.45	0.74

*CHUY* Hospital and University Center, *HCY* Yaoundé Central Hospita, *H* Military Hospital, *HP* Police Hospital, *HE* Efoulan District Hospital, *HIV* Human immuno-deficiency virus, *CI* Confidential interval, *OR* Odd ratio

### Prevalence of *Clostridium difficile* in Yaoundé, Cameroon

Our results showed that CD prevalence was 27.3% and the proportions of GDH and sub types of CD were 37.8 and 55.0% respectively. Our findings documented that hospitalization was significantly associated with CDI-based on gender, age, antibiotic use and hospitalization in selected hospitals in Cameroon. Interestingly, based on univariate analysis, 4.16% of inpatients using cortocoids were positive to *C. difficile against* 0.5% not using this drug. Although the difference was not statistically significant (*P* = 0.06). Likewise, 21.4 and 44.8% of inpatients on antibiotics and quinolones were *C. difficile* reservoirs against 28.98 and 26.9% not using antibiotics with not statistically significant difference respectively (*P* = 0.26; *P* = 0.36) (Table 3).

**Table 3:** Multivariate analysis of risk factors associated with CDI reservoir or prevalence based on antibiotics

Variables	Adjusted ***OR***	95% ***CI***	***P***-value
**Hospitalization**			
Not	1		
Yes	0.99	1.18–4.00	0.01
**Age**			
Yes	0.98	0.97–1.00	0.06
**Use of vancomycine**			
Not	1		
Yes	4.56	0.71–29.38	0.11
**Use of aminoside**			
Not	1		
Yes	2.74	0.32–23.24	0.36
**VIH**			
Not	1		
Yes	0.96	0.55–1.78	0.98
**Use of quinolone**			
Not	1		
Yes	0.39	0.07–2.23	0.29
**Use of corticoids**			
Not	1		
Yes	1.32	0.18–9.6	0.78
**Use of imidazole**			
Not	1		
Yes	0.89	0.31–2.61	0.84
**Use of cephalosporins**			
Not	1		
Yes	0.7	0.16–2.75	3.00
**Use of penicillin**			
Not	1		
Yes	0.94	0.3–2.94	0.92

*CDI Clotridium difficile* infection, *CI* Confidential interval, *OR* Odd ratio

### Prevalence of diarrheal cases in *Clostridium difficile i*npatients

Our results showed that 11.1% of inpatients with diarrhea were females against 8.3% in males; with no statistically significant difference (*P* = 0.69). Nonetheless,11.9% of hospitalization had diarrhea and about 5.6% of inpatients with toxin A had diarrheaagainst 12.5% in those with both toxin A et B; but were not statistically significant (*P* > 0.05) based on univariate analysis (Table 4). While analysis the risk factors of diarrhea susceptibility among CDI, we found that only imidazole treatment was significantly associated with diarrheal diseases occurrence in patients with *C. difficile* infection (a*OR* = 29.0; 95% *CI*: 3.0–281.24; *P* = 0.004) (Table 5).

**Table 4:** Prevalence of diarrheal cases in *Clostridium difficile* inpatients according to sex, hospitalization, HIV and bacteriotoxin

Variable	Total (***n***)	No diarrhaoe ***n*** (%)	Diarrhaoe ***n*** (%)	***P***-value
**Sex**				
Male	36	33(91.7%)	3(8.3%)	0.69
Female	36	32(88.9%)	4(11.1%)	
**HIV**				
Negative	47	43(91.5%)	4(8.5%)	
Positive	25	22(88%)	3(12.0%)	0.63
**Hospitalization**				
Not	13	13(100.0%)	0(0.0%)	
Yes	59	52(88.1%)	7(11.9%)	0.97
**Bacteriatoxin**				
No toxin	48	42(87.5%)	6(12.5%)	
Toxin A	18	17(94.4%)	1(5.6%)	0.43
Toxin A + B	6	6(100.0%)	0(0.0%)	0.98

*HIV* Human immuno-deficiency virus

**Table 5:** Multivariate analysis of risk factors of diarrhea in *Clostridium difficile reservoirs*

Variables	Adjusted ***OR***	95% ***CI***	***P-***value
**Imidazole**			
Not	1		
Yes	29.06	0.97–1.00	0.004
**β-lactams**			
Not	1		
Yes	0.90	0.95–1.00	0.95
**Quinolone**			
Not	1		
Yes	3.15 × 10^-5^	0.97–1.00	0.98

*CI* Confidential interval; *OR,* Odd ratio

Overall, our findings showed that 26.3% of inpatients took imidazole group of drugshad CD infection whereas 27.45% inpatients did not have, but the difference was not statistically significant (*P* = 0.91) based on univariate analysis. Vancomycine and aminosides were fully sensitive to *C. difficile*, whereas 27.8% of inpatients has CDI, with no significant difference (*P* = 0.97) based on univariate analysis (Table 5).

## Discussion

Our finding showed a prevalence of *C.difficile* infection of 25.1%, mainly within the age group of 19 to 55 years old in selected five hospitals in Yaoundé, Cameroon. The sex ratio was 1.07 with predominance 55% males may be due to the selection of military and police forces hospitals with relatively similar *C. difficile* prevalence in males and females of 26.45 and 28.28% respectively.

Our results are in contrast with Zilberberg et al., 2008 findings, of *C. difficile e*xposure in patients > 85 years old [9]. Similarly, Barbut & Petit, 2001, reported that the population incriminated was > 65 years old [10]. The difference may be explained by old age population > 65 years old. Our findings were closer to Dial et al. that reported that women were twice more exposed to *C. difficile* than men [11].

Our study had more inpatients from CHUY (94), but *C. difficile* infection prevalence was higher in HM (32.4%). Johnson et al., 1990 and Barbut & Petit., 2001 reported association of risk factors enabling transmission dynamics of spores to current use disinfectants and antiseptics, selective pressure of antibiotics in inpatients and promiscuity, hospitalization environment favoring bacteria propagation [12]. Regarding antibiotics uses, 40.0% of CDI was associated with beta-lactamases use. These finding are consistent with previous studies linking CDI to large spectrum antibiotics use [11, 12]**.**

Our finding documented a prevalence of *C. difficile* of 27.33%. This indicates an increase of over three folds to Barbut & Petit that reported 8% over 100 000 patients [3]. Our findings can be explained by the growing risk factors including poor sanitation and hygiene, poor health facilities and lack of environmental management and contaminants favoring *Clostridium difficile* porulation [13].

Excess use of antibiotics (quinolone) have a significant impact on digestive tract flora and alter the digestive system biotic (a*OR* = 3.39; 95% *CI* = 1.00–11.34; *P* = 0.05), similar to M. Ingle et al. in India that reported the use of antibiotics act severely on digestive system (*P* = 0.06). Consumption of corticoids and anticancer drug use appear to weaken the body system or immune-depressive factor rendering the body more susceptible [13].

Toxins A and B secretion were80 and 7.31% respectively. Nowadays, morbidity and mortality resulting from infectious diseases associated with *C. difficile* have change considerably due to subtype’s virulence variations, use of antibiotics. Both toxins have a similar sequence of amino acid of 63% and are member of large family of glucosylant clostridium toxin, which are pro-inflammatory monoglucosyltransferases, cytotoxic enterotoxic effects in human gut. Within the host cells, both toxins catalyzed the transfer of glucose leading to cell death or apoptosis. However, the role of these toxins in CDI is still poorly understood. Lyras et al. showed that toxin B is essential in CDI severity [14]. Thus, the concentration of toxin B of 7.31% of *C. difficile* reservoir remains worrisome.

Univariate analysis showed that hospitalization was the principal risk factor associated with *C. difficile* reservoir or infection, thus is consistent with Barbut et al. that reported an incidence of digestive infectious diseases linked to *C. difficile* reservoirs varying from 1 to 10 per 1000 inpatients admitted [15]. Similarly, risk factors of CD reservoirs including use of corticoids (*P* = 0.02; *OR* = 14.09) and quinolones (*P* = 0.05; *OR* = 3.39) were statistically significant and consistent with Barbut et al. [15]. Equally, Barbut & Petit reported that consumption of antibiotic altered digestive tract [10] and Zilberberg et al. showed that fluoroquinolones were linked in digestive tract *C. difficile reservoirs* [9]. Multivariate analysis showed that imidazole family was responsible of diarrhea in inpatients and statistically significant association with *C. difficile of* 60% in inpatients having used this antibiotics (*P* = 0.036, a*OR* = 29.06). This result is contrary to Natalia et al. in Russia in 2018 [16], where the β- lactamases group and quinolones were linked to patients with *C. difficile*. 11.86% of hospitalized patients had diarrhea but not statistically significant univariate analysis; this result is lower that Sachu et al. [17] of 21.7% inpatients admission with diarrhea. The variation may be due to population size, inpatient admission and poor hygiene and sanitation. However, 5.56% of inpatients had toxin A having diarrhea, but no statistically significant (*P* = 0.43), lower than N. Salle, 2009 of 10–25% in France [17]. Sachu et al. [18] reported statistically significant relationship between toxin A having diarrhea. This indicates that the presence of toxins in CDI patients or reservoirs is a risk factor of diarrhea, but our study found no significant association and most inpatients were on antibiotics origin of diarrhea related symptoms.

Exposure to antibiotics mainly β-lactamases and quinolones are known as risk factors of diarrhea illnesses associated with *C. difficile* infection [19, 20], but no statistically significant(*P* = 0.78, a*OR* = 0.9, a*OR* = 3.15 × 10^-5^) based on multivariate analysis. Ingle et al. reported an association with antibiotic use in India (*P* = 0.067) [21].

Limitations of the study included the sensitivity and specificity of kits varied from 70 to 80 and 95% respectively, correlation between antibiotic use and CDI change, lack of routine hospital infection control and diagnostic assay data. In addition of false negative and false positive samples were documented. Likewise, the serological shortage unables us to establish the linkage between CD reservoirs and CDI to HIV prevalence and complications.

## Conclusions

The documented high prevalence of *C. difficile* infection of 27.83% indicates that it is one of the primary source/cause of entero-nosocomial diarrheal diseases in adults’ populations. The increasing incidence and prevalence of CDI severity calls for collaborative commitment and investment of all stakeholders including the vulnerable communities in strengthening laboratory surveillance through early toxins detection in feces or blood samples for evidence-based hospital infection prevention and control best practices capacity building, in ensuring quality care delivery and an appropriate antibiotics prescription, safety precautions and outcomes.

## Data Availability

All data are available upon request.

## Abbreviations

WHO: World Health organisation
CDI: Clotridium difficile infection
CD: Clotridium difficile
HIV: Human immuno-deficiency virus
CHUY: University Hospital Center, Yaoundé
HCY: Central hospital, Yaoundé
HM: Military hospital
HP: Police Hospital
HE: Efoulan hospital
*CI*: Confidential interval
*OR*: Odd ratio
